# Vaccines and Immunotherapeutics for the Treatment of Malignant Disease

**DOI:** 10.1155/2010/697158

**Published:** 2010-09-26

**Authors:** Joel F. Aldrich, Devin B. Lowe, Michael H. Shearer, Richard E. Winn, Cynthia A. Jumper, Ronald C. Kennedy

**Affiliations:** ^1^Department of Microbiology and Immunology, Texas Tech University Health Sciences Center, 3601 4th Street, MS 6591, Lubbock, TX 79430, USA; ^2^Department of Internal Medicine, Texas Tech University Health Sciences Center, 3601 4th Street, MS 9410, Lubbock, TX 79430, USA

## Abstract

The employment of the immune system to treat malignant disease represents an active area of biomedical research. The specificity of the immune response and potential for establishing long-term tumor immunity compels researchers to continue investigations into immunotherapeutic approaches for cancer. A number of immunotherapeutic strategies have arisen for the treatment of malignant disease, including various vaccination schemes, cytokine therapy, adoptive cellular therapy, and monoclonal antibody therapy. This paper describes each of these strategies and discusses some of the associated successes and limitations. Emphasis is placed on the integration of techniques to promote optimal scenarios for eliminating cancer.

## 1. Introduction

As cancer progresses toward the leading cause of death in the Unites States, physicians and biomedical scientists continue to explore novel therapeutic strategies outside the current standard of treatment. Despite the successes of surgery, radiation, chemotherapy, and a combination thereof in limiting the progression of malignant disease, these treatment methods often fail to elicit complete tumor remission and are associated with some debilitating side effects. In recent years, much attention has been paid to immunotherapy, which attempts to direct the protective capacity of the immune system toward eliminating malignancies. Harnessing the immune system to treat malignant disease is a powerful tool, not only due to the specificity of the immune response, but also due to the potential for establishing long-lasting tumor immunity via the capacity to exhibit memory. The ability of the immune system to destroy tumorigenic cells was first proposed by Macfarlane Burnet in the 1950s [1]. Some years later, Burnet coined the term “immune surveillance” to describe the function of the immune system in eliminating transformed cells both before and after tumor formation [2]. A seminal study conducted by Shankaran and colleagues in 2001 confirmed the importance of certain immune components in limiting the formation of tumors in experimental animals. In this study, immunocompromised mice were found to be significantly more susceptible to spontaneous and carcinogen-induced primary tumor development than immunocompetent mice [3]. The critical role of the immune system in minimizing malignancies engenders profound sequelae in the human situation as well. Certain immunodeficiency disorders, including AIDS, are strongly associated with an increased risk of cancer [4]. Additionally, the formation of tumors in immunosuppressed organ transplant patients and among individuals receiving stem-cell transplants has been well documented and represents a major obstacle to the long-term success of these procedures [5]. Collectively, such findings provide an impetus for continual investigation of the therapeutic potential of antitumor immune responses.

Immunotherapeutic strategies can be categorized broadly into two groups: active immunotherapy and passive immunotherapy. Establishing active immunity against tumors is a promising but inherently difficult task, and necessitates a keen understanding of the multiple immunosuppressive mechanisms that the tumor microenvironment may exploit. According to Waldmann, maximizing the efficacy of active immunotherapy will require a thorough investigation of the appropriate target antigens; the optimal interactions between lymphocytes, antigen-presenting cells (APC), and antigens; and the obstruction of negative immune regulation [6]. Although this immunotherapeutic strategy holds the potential for establishing long-lasting tumor immunity, the dissolution of immune tolerance to prospective cancer antigens remains a challenging and controversial process. The possibility of eliciting rampant autoimmunity in the wake of tumor reactive lymphocytes remains a key concern in the ultimate utility of active immunotherapy, particularly when this therapy is used in combination with other immunostimulatory techniques [7, 8]. 

 Passive immunotherapy using clonally expanded tumor-specific T cells represents a different approach to manipulating components of the host's immune system to target cancer. Unlike active approaches, tumor-specific lymphocytes are expanded *ex vivo*, allowing for more direct manipulations of the prospective immune effectors. As with active immunotherapy, however, the possible long-term effects of harboring self-reactive lymphocytes warrants further assessment. Additionally, passive immunotherapy using monoclonal antibodies (MAbs) and immunoglobulin (Ig)-fusion proteins is a rapidly emerging technology that holds great potential for effectively treating malignant disease. The increasing incidence of MAb therapy in the treatment of cancer and other diseases firmly establishes the legitimacy of such molecules as effectual and specific anticancer agents [9]. A total of nine MAbs and modified Ig molecules have been approved by the FDA for use in cancer patients, and many more are in the process of clinical trials. Despite the enthusiasm for this type of therapy, several key challenges still remain for optimizing the efficacy of these artificial immune effectors. Such challenges include minimizing the induction of host-neutralizing antibody responses and curtailing the residual cytotoxicity of some Ig-fusion molecules. Additionally, both vaccination and MAb approaches to tumor immunotherapy may encourage the generation of tumor cells that evade immune recognition. In accordance with the process of “immunoediting,” tumor cells that bear antigenic targets for vaccination or MAb therapy are subject to destruction; however, tumors may compensate by expanding populations of antigenically undetectable tumor cells [10, 11]. These “immune escape variants” arise due to selective pressures imparted on the tumor microenvironment by antigen-specific immunotherapies, and subsist via the strategic masking of antigens that are recognized by the antitumor immune response. 

## 2. Vaccination As an Immunotherapeutic Tool

### 2.1. Identification of Appropriate Tumor Antigens

Given the historic success of active immunization in protecting against infectious microbial diseases, many researchers are attempting to apply vaccination approaches to cancer immunotherapy. Indeed, several prophylactic vaccines have been generated against viral infectious agents that are also causative for certain human cancers. FDA-approved vaccines against hepatitis B virus (HBV) and human papilloma virus (HPV) are associated with protection against HBV-induced liver cancer and HPV induced cervical carcinomas, respectively. This clearly demonstrates that vaccines can be produced to prevent human malignancies. There are several vaccine modalities currently under investigation, including protein/peptide vaccines, ex-vivo loaded dendritic cells (DCs), DNA vaccines, and recombinant viral/bacterial vectors expressing particular tumor antigens. Additionally, prime-boost vaccine strategies seek to optimize the immune response by combining two or more of these modalities into a single treatment regimen. Common prime-boost strategies include primary immunization with plasmid DNA and subsequent immunizations with recombinant protein or viral vectors, although considerable variations on this theme abound within the literature [12].

The ultimate intention of immunization is induction of a tumor-specific immune response, thus the identification of appropriate tumor antigens remains a key concern for each of these vaccine strategies. Among the various categories of candidate antigens, tumor-specific antigens represent ideal targets, as these molecules are expressed exclusively on tumor cells. Examples of tumor-specific antigens include the products of mutated oncogenes and altered tumor suppressor proteins. One such tumor suppressor protein is p53, which plays a critical role in regulation of the cell cycle and is a target of some oncogenic viral proteins, including Tax from human T-cell lymphotropic virus-1 (HTLV-1) [13] and large T antigen (Tag) from simian virus 40 (SV40) [14]. Despite numerous reports of detectable humoral responses against p53 in cancer patients, the protection afforded by such responses appears to be minimal [15]. Additionally, the limited propensity for oncogenic mutants of normal cellular genes to promote the generation of protective cytotoxic T lymphocyte (CTL) responses presents a major obstacle to the exploitation of these antigens [16]. Within the last decade, several tumor-specific self-antigens that are recognized by CTLs have been identified (including CDK-4, *β*-catenin, and Caspase-8), and show potential for incorporation into cancer vaccines [17].

In addition to tumor-specific self-antigens, viral oncoproteins represent a unique class of tumor antigen that, during the course of viral infection, may be expressed primarily on transformed cells and infected cells harboring an increased neoplastic potential. A study performed by Duraiswamy and colleagues in 2003 provided convincing evidence of the ability for a polyepitope vaccine directed against the latent membrane protein 1 (LMP1) of Epstein-Barr virus (EBV) to provide immunity against aggressive tumors expressing LMP1 in mice [18]. Importantly, the tumor immunity evoked in this model was observable both in a prophylactic setting and in a therapeutic vaccine scenario. Such findings continue to compel researchers to investigate vaccination schemes that target viral oncoproteins as tumor-specific antigens. Indeed, the efficacy of both SV40 Tag recombinant protein and SV40 Tag DNA vaccines in protecting mice against Tag expressing tumors has been well documented by our laboratory [19]. While only a few viruses have been directly implicated in the generation of tumors in humans (namely HTLV, EBV, and HPV), the pathology associated with certain other viruses, including human immunodeficiency virus (HIV), HBV, and hepatitis C virus (HCV), may promote the development of tumors in some individuals. Certain viruses may also act synergistically to provoke tumorigenesis; for example, coinfection with Kaposi's sarcoma-associated herpesvirus and HIV often results in the formation of disseminated blood vessel tumors. In addition to viral pathogens, gastric inflammation induced by the bacterium *Helicobacter pylori* has been suggested to encourage the growth of local tumors. Accordingly, vaccines that eliminate these oncogenic and prooncogenic microbes may provide protection against malignant disease prior to the formation of tumor foci. 

 Unfortunately, many types of cancer do not express universally recognized antigens that are associated exclusively with tumor cells. Investigators must therefore explore the use of other antigens that are expressed differentially on normal and cancerous cells. Various categories of tumor associated antigens (TAAs) have been described, including overexpressed self-antigens, differentiation antigens, and antigens from immune privileged sites (cancer/testes antigens) [17]. The first TAA to be identified was MAGE-1, which is an antigen expressed in tumor cells and germ cells and is prone to recognition by CTLs [20]. The absence of MAGE expression in most normal adult tissues (including liver, muscle, skin, lung, brain, and kidney), and the relative abundance of this antigen in tumors and germline tissues (e.g., testis, placenta, ovary), qualifies MAGE as a classic cancer/testes (CT) antigen. Moreover, vaccines that target CT antigens are unlikely to cause collateral tissue destruction, as normal adult cells are not transcriptionally active for CT antigens and germ cells lack the necessary machinery for antigen presentation to the immune system. Other major TAA categories include differentiation antigens, of which the melanocyte proteins tyrosine and MART are examples, and overexpressed self-antigens, of which the common breast cancer antigen HER-2/neu (ErbB2) is an example. These antigens are thought to be expressed by such a small group of cells and/or in such limited quantities, that the immune system fails to induce tolerance to these self proteins. Several prospective self-antigens have thus been identified for use in cancer immunotherapy [21], with some of the more common antigens listed in Table 1.

### 2.2. Vaccination Strategies

Although protein/peptide vaccination with purified antigen plus adjuvant has long served as an effective vaccine strategy in the prevention of microbial disease, recent advances in the field of vaccine development may favor the use of DNA-based or APC-based vaccines in the treatment of malignant disease. Despite numerous studies that have demonstrated the antitumor potential of conventional recombinant protein vaccination, this vaccine modality may curtail antigen presentation through the major histocompatibility complex (MHC) class I pathway and elicit a predominantly humoral immune response [22]. Since CTLs are commonly thought to comprise the major effector cell type in tumor immunity, vaccination methods that enhance cell mediated immune responses may prove optimal for use in cancer immunotherapy.

In the early 1990s, Wolff and colleagues reported that transgene expression in mice could be accomplished upon direct injection of naked plasmid DNA into mammalian muscle tissue [23]. Subsequently, Ulmer and colleagues used another murine model to demonstrate the utility of DNA vaccines as a preventative against heterologous influenza virus infection [24]. Translational studies targeting HIV and malarial antigens commenced in the late 1990s, and soon established the safety of this vaccination scenario in humans [25–27]. Within the last decade, experimental DNA vaccination in dogs has demonstrated the efficacy of this vaccine modality in prolonging survival time within the context of aggressive malignant disease. In one important study, dogs suffering from canine malignant melanoma were immunized therapeutically with plasmid DNA encoding human tyrosinase, which is approximately 91% identical to canine tyrosine [28]. The median survival time of dogs in this study was 389 days; substantially higher than the <2-3 month survival time observed in historical, stage-matched controls. Positive clinical outcomes appeared to correlate with the induction of antibody responses to canine tyrosinase in some animals, although a potential role for T cell mediated immunity in this system is still under assessment [29].

The ever-growing compendium of literature involving DNA vaccines reveals a number of key advantages for this emerging vaccine modality. In contrast to protein/peptide vaccines, DNA vaccines have adequately demonstrated the capacity to mobilize both the cell mediated and humoral arms of the immune system in animal models [8, 22, 30]. In addition, the production of plasmid DNA is less complicated than that for protein, providing a crucial economic incentive for the use of this vaccine modality. The biochemical properties of DNA also permit extensive handling at room temperature, diminishing the need for maintenance of a stringent cold chain during the distribution process [31]. Perhaps the most attractive feature of DNA vaccines is the option for convenient manipulation of the encoded immunogen's nucleotide sequence. This attribute permits the immediate generation of antigen-cytokine fusion molecules and other immunostimulatory complexes that might otherwise be challenging to construct. In the late 1990s, Song and colleagues used a murine model to survey the helper T cell (Th1) versus Th2 proclivity of various ovalbumin cytokine DNA constructs, and provided evidence that significant skewing of antigen specific immune responses can occur with the use of antigen-cytokine fusion molecules [32]. Additionally, multiple antigenic targets or multiple independent cytokines can be incorporated into a single DNA vector. As with the aforementioned fusion molecules, studies conducted in mice have indicated that the nature of the resultant immune response is highly dependent on the immunostimulatory penchant of the cytokines utilized in the vaccine [33]. A final potential advantage of DNA vaccination is that transgene expression is thought to occur over an extended period of time, perhaps obviating the potential need for an abundance of repetitive booster vaccinations. Moreover, the protective potential of DNA vaccination in animals has been substantiated by reports of DNA constructs mediating long-term tumor immunity, particularly when utilized in a prime-boost scenario or in combination with immunomodulators [34, 35].

A major challenge facing DNA vaccines, however, is the elicitation of a robust immune response in the human clinical setting. Translational applications of DNA vaccines have consistently suffered from low immunogenicity; consequently, several unique prime-boost strategies have been developed to amplify the immune response. A multitude of distinct delivery methods exist for DNA vaccines (including intramuscular injection, biolistic gene gun delivery, modified viral vectors, etc.), allowing for the creation of a large repertoire of heterologous prime-boost scenarios. As mentioned previously, heterologous prime-boost scenarios generally focus on the incorporation of distinct vaccine modalities into a single treatment regimen; however, another application of this technique incorporates a single vaccine modality with multiple distinct delivery methods into a single treatment regimen. One particularly successful embodiment of this strategy in murine models involves priming of the immune system with an intramuscular or intradermal injection of plasmid DNA, followed by electroporation of the homologous DNA in booster immunizations [36, 37]. In addition, the ability of CD4+ helper T cells to enhance CTL activity has prompted investigations into prime-boost scenarios that utilize different DNA constructs aimed at engaging distinct presentation pathways [38]. In the human clinical situation, Todorova and colleagues showed that specific antibody could be induced by vaccinating with alternate injections of a prostate specific membrane antigen (PSMA)-expressing adenoviral vector and plasmid DNA encoding PSMA and CD86 in a majority of participants [39]. As indicated previously, cytokines and other immunostimulators can be incorporated directly into the DNA vector to improve immunogenicity. Experience with animal models suggests that prudent selection of these companion molecules may allow researchers to promote induction of a predominantly cell mediated or humoral immune response to the encoded tumor antigen [32, 33].

APC-based vaccines represent another popular vaccine moiety in cancer research. With this approach, DCs are harvested from the patient, pulsed with tumor antigens or transfected with genes encoding these antigens, and readministrated to the patient. As with DNA vaccination, this vaccine strategy has the potential to augment presentation through the MHC-class I pathway and subsequently drive the expansion of tumor-specific CTLs. In translational studies with melanoma patients, DC vaccines have demonstrated a keen ability to elicit detectable immune responses; however, such responses often fail to elicit substantial clinical responses [40]. As it is often difficult to discern the relative contributions of DCs and effector T cells in these situations, a thorough investigation of the *in vivo* interactions between these immune cell populations may be required before a complete understanding of DC function in tumor immunity can be elucidated [40].

One aspirant application of DC-based immunotherapy includes the recently reported Sipuleucel-T immunotherapy (Provenge^®^) developed by Dendreon^*™*^. With this strategy, peripheral blood mononuclear cells, including DCs, are harvested from the patient and activated *in vitro* with prostatic acid phosphatase, a differentiation antigen, linked to granulocyte-macrophage colony-stimulating factor (GM-CSF). In a clinical trial with 225 patients experiencing advanced metastatic androgen independent prostate cancer, Sipuleucel-T immunotherapy was able to extend survival by ~4 months [41]. In April 2010, Sipuleucel-T immunotherapy was approved by the FDA for the treatment of asymptomatic or minimally symptomatic metastatic, castrate-resistant prostate cancer. This autologous cellular immunotherapy represents the first therapeutic cancer vaccine to acquire FDA approval, and provides encouragement for the continued development of similar vaccine strategies. 

**Figure 1:**
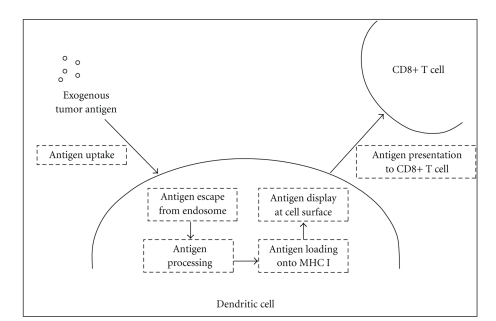
General mechanism of tumor antigen cross-priming to CD8+ T cells. In the process of cross-priming, exogenous tumor antigen (which may be released from tumor cells via apoptosis, necrosis, or immune-mediated damage) is endocystosed by the DC. Antigen then escapes from the endosome and is processed and loaded onto MHC class I alongside cytosolic antigens. Peptide-loaded MHC class I molecules are ultimately transported to the cell surface, where they may encounter and activate CD8+ T cells through interactions with the T cell receptor [42].

 In the protein/peptide pulsed DC scenario, vaccine efficacy may be largely dependent on the DCs' ability to shuttle exogenous antigen through the MHC class I pathway, enabling cross priming of an array of TAAs to CD8+ CTLs (Figure 1). Interestingly, recent reports indicate that the efficiency of cross presentation is at least partially dependent on the length of the antigenic peptides. In a study performed by Faure and colleagues, shorter peptides were more efficiently presented to CD8+ T cells after incubation with DCs; however, after an extended chase period in the absence of peptide, longer peptides were more efficiently presented [43]. The results from this study indicate that peptide size is an important consideration in any DC-based elicitation of long-term tumor immunity. In the genetically modified DC scenario, presentation through the MHC class I pathway can be accomplished directly by processing of endogenous antigen within the DC. Interestingly, bacterial plasmids commonly utilized as DNA backbones may have self-adjuvanting capabilities, as unmethylated CpG regions have been shown to bind Toll-like receptor 9 and stimulate innate immunity [44, 45]. In one relevant setting, Yang and colleagues used a transgenic mouse model to demonstrate that *ex vivo *stimulation of DCs with CpG-containing oligodeoxynucleotides plus antigen could break CD4+ regulatory T cell (Treg)-mediated tolerance of CD8+ T cells to tumors [46]. In numerous animal models, immunogenic enhancement of cell-based vaccines has been accomplished by using novel combinatorial techniques, such as *ex vivo* transfection of DCs with lentiviral vectors that harbor antigen-encoding DNA [16, 47–49]. Aside from conventional tumor-focused approaches, experimental APC-based vaccines aimed at treating chronic viral infections, such as HCV infection [50], may indirectly reduce the onset of virally induced cancers.

## 3. Cytokine-Based Immunotherapy

In addition to active immunization, cytokine-based therapies embody a direct attempt to stimulate the patient's own immune system to reject cancer. A number of strategies exist for introducing cytokines into cancer patients, including the incorporation of cytokine genes into DNA vaccines and the systemic administration of immunostimulatory molecules. If cytokines are incorporated into a DNA vector, direct transfection of autologous tumor cells denotes a possible treatment option. This might allow for the localization of cytokines to the tumor site, promoting the expansion of neighboring immune cells and possibly abrogating the need for additional treatment with antigenic peptides. Indeed, antitumor responses directed against genetically modified tumor cells have been documented in a number of murine models exploring a number of prospective immunotherapeutic cytokines. These models have generally focused on tumor immunity mediated by cytokines that promote differentiation of the Th1 subset of CD4+ T cells, including IL-12 [51], IL-18 [51], IL-15 [52], IL-21 [53], IL-23 [54, 55], and IL-27 [56], among others. Before this approach can be utilized to its maximum potential, however, a precise understanding of the resultant tumor microenvironment and of the recruited immune cells is necessary. For example, the incorporation of certain proinflammatory cytokines (e.g., IL-1, IL-6) into the immunosuppressive tumor microenvironment may encourage the generation of somewhat enigmatic T cell populations, including Th17 cells. While there is compelling evidence that Th17 cells may beget tumor immunity by recruiting tumor reactive CTLs into the tumor site [57], it is important to consider that this cell population was initially described within the context of autoimmunity [58–61]. Further assessment of the pathologic versus protective functions of such cell subsets should be performed prior to their intentional or unintentional employment in tumor immunity.

At the opposite end of the cytokine-based treatment spectrum is the systemic administration of cytokines. Utilization of cytokines in this manner presumably stimulates the proliferation of certain immune cells in a non-specific manner, expanding immune cell populations that may include protective tumor-infiltrating lymphocytes (TILs). The efficacy of intravenously introduced IL-2 in patients with metastatic melanoma or renal cell cancer has been documented in a number of studies [62, 63], but discerning the appropriate dosage and treatment schedule with immunostimulatory cytokines remains a time-consuming and trying process. Alternatively, murine models indicate that the use of cytokine therapy in combination with other treatment modalities, including CpG-containing peptide vaccines [64], may obviate the need for high-dose administration of cytokines and lower the incidence of treatment-associated sepsis. 

 As mentioned previously, IL-2 is a cytokine commonly used in studies of tumor immunotherapy. IL-2 has been shown to subvert cancer progression in some patients [62, 63]; however, the T cell mediated production of proinflammatory cytokines in response to this cytokine can result in severe toxicity and limits its use as a singular treatment modality. In addition, IL-2 has been implicated in eliminating self-reactive T cells by a process known as activation-induced cell death, which may obscure the antitumor effects of T lymphocytes produced within the tumor microenvironment [65]. Despite these setbacks, intravenously administered IL-2 endures as an FDA approved immunotherapeutic treatment option for patients with metastatic melanoma or renal cell carcinoma. Another commonly studied cytokine in cancer immunotherapy is GM-CSF, which acts predominantly by promoting the recruitment and maturation of DCs. The antitumor effects of GM-CSF have been documented in numerous studies, often in conjunction with vaccines or other immunotherapeutic strategies. One notable study conducted in 2002 surveyed various immunostimulatory molecules for their ability to enhance antitumor immune responses across multiple murine models [66]. In this study, GM-CSF consistently proved to be the most potent of the tested products. In addition to IL-2 and GM-CSF, several other cytokines, including various interleukins, interferons (IFNs), and tumor necrosis factor, have been investigated for their immunotherapeutic potential. As is the case with IL-2, most of these cytokines are limited by some degree of systemic toxicity. In spite of this, a recent study performed with an experimental renal carcinoma model suggests that sub-optimal doses of combined IL-21 and IFN-*α* can mediate antitumor immunity without the appearance of adverse side effects [67].

## 4. Adoptive Cellular Therapy

In some cancer patients, passive immunization with immune effectors may constitute a more practical or desirable approach to immunotherapy than active immunization. One such treatment method is adoptive cellular therapy, which utilizes modified components of the patient's own immune cell repertoire to promote rejection of established tumors. In adoptive cellular therapy, peripheral blood leukocytes or TILs are harvested from the patient, expanded *in vitro* with antigen or stimulatory cytokines, and injected back into the patient. The culturing of NK cells in the presence of IL-2 generates lymphokine-activated killer cells, which, in conjunction with CTLs, are capable of mounting an aggressive immune response to tumor cells. In 1994, Rosenberg and colleagues demonstrated the utility of adoptive cellular therapy in metastatic melanoma patients by transfer of autologous TILs in combination with high-dose IL-2 [68]. The response rate of the 86 patients treated in this clinical study approached 34%, although responses were generally characterized by a short duration. A few years later, Yee and colleagues performed an assessment of adoptive cellular therapy in metastatic melanoma patients by selecting and expanding TAA-reactive CTL clones from peripheral blood mononuclear cells [69]. Although regression of individual metastases was reported, the results from this study failed to yield objective responses according to RECIST criteria [70]. Interestingly, lymphodepletion with chemotherapeutic drugs prior to the onset of adoptive cellular therapy has led to vast improvements in the efficacy of this immunotherapeutic modality. In a study performed by Dudley and colleagues in 2005, over 50% of metastatic melanoma patients experienced objective reponses according to RECIST criteria upon treatment with lymphodepleting chemotherapy followed by adoptive transfer of tumor reactive lymphocytes [71]. The prevailing logic behind this combinatorial approach is that endogenous toleragenic host lymphocytes compete with the transferred cells for homeostatic cytokines, and must be depleted prior to adoptive cellular therapy in order to achieve optimum antitumor responses. Additionally, activation and loading of endogenous DCs may be enhanced by the milieu of tumor antigens released upon the administration of chemotherapeutic agents [72]. 

 Aside from the standard expansion protocols for effector and antigen presenting cells, adoptive cellular therapy allows for direct ex-vivo manipulation of these immune cell populations. Extraction of a general pool of lymphocytes may complicate the process of identifying and expanding certain antitumor lymphocytes *in vitro*, thus some researchers have explored the introduction of specific T cell receptors (TCRs) directly into these cells. This technology can be used to rapidly generate a band of chimeric T cells reactive towards a particular antigen; a strategy that may prove particularly useful for patients with nominal quantities of TILs. Additionally, the use of novel molecules such as “TCR-like” Fab fragments may avert problems associated with unintentional pairing of endogenous and introduced TCR chains [73]. In any case, the efficacy of genetically modified lymphocytes in combating tumor outgrowth has been observed in the clinical setting [74]. In addition to genetic engineering of TCRs, the production of modified antigenic peptides may beget enhanced T cell induction for use in adoptive cellular immunotherapy [75]. Despite these exciting and promising new technologies for adoptive cell transfer, the practicality of extending this time-consuming and expensive procedure to the general public remains to be determined.

## 5. Monoclonal Antibodies As an Immunotherapeutic Tool

The use of MAbs and antibody conjugates to treat malignant disease has held the interest of the scientific community since the time of Ehrlich [76]. Moreover, the FDA's approval of nine MAbs for the treatment of cancer has placed a promising outlook on the expansion of this therapeutic modality in the near future. Akin to immunotherapeutic vaccines, MAbs are designed to target specific antigenic sequences associated with tumor cells; however, these antigenic targets generally must reside on the surface of the cell in order for antibody therapy to be effective. Additionally, MAbs and cancer vaccines share the potential for extensive molecular modification to improve the effectiveness of immunotherapy. Unlike cancer vaccines, supplementary molecules fused to MAbs need not be peptides, as various toxins and radioisotopes can also be effectively conjugated to the Fc region of the Ig molecule. Experimental murine models have indicated that antibody dependent cell-mediated cytoxicity (ADCC) is an important effector mechanism of antitumor antibodies [77]; however, additional methods of tumor eradication may include opsonization followed by phagocytosis and activation of complement. One potential advantage of MAbs over cancer vaccines is that the number of circulating immune effectors in the patient can be raised simply by increasing the dosage, a feat that is not always easily achievable in cancer vaccines. At the very least, MAb therapy may serve as an effectual alternative to cancer immunization, particularly in situations where the development of autoimmunity is a concern.

Table 2 provides a list of the MAbs approved for therapeutic use in cancer patients by the FDA. Of the nine molecules, two represent radioimmunoconjugates (ibritumomab tiuxetan and ^131^I-tositumomab), and one (gemtuzumab ozogamicin) represents a cytotoxin-conjugated antibody. These molecules are unique in that the direct antitumor effect elicited by the accompanying radioisotope or cytotoxin trivializes the need for engagement antibody Fc regions for ADCC or other natural effector mechanisms. The efficacy of such modified MAbs over an extended time period may be limited, however, due to the potent cytotopathic effects of residual molecules circulating through the body, and the induction of neutralizing immune responses to the conjugated toxin and/or non-humanized antibody regions. Additionally, animal models indicate that antiidiotype responses to the MAb used for treatment can also potentially neutralize the effectiveness for targeting the tumor [78]. Many of the MAbs, including trastuzumab and rituximab, consist of humanized or chimeric Fc regions and, for both of these therapeutic agents, indications of a role for ADCC have been reported [79, 80]. While the antigenic targets for most of the approved MAbs are characteristic tumor cell markers (including the B cell activation marker CD20, the myeloid transmembrane receptor CD33, and the lymphocyte surface antigen CD52), three of these therapeutic agents (cetuximab, bevacizumab, and panitumumab) target molecules directly implicated in tumor cell outgrowth. In some cancers, tumor formation is dependent on aberrant expression of epidermal growth factor receptor (EGFR), which serves as an antigenic target for cetuximab and panitumumab. Similarly, bevacizumab targets vascular endothelial growth factor (VEGF), which is a secreted factor that promotes angiogenesis within the immediate vicinity of the tumor. These latter MAb treatment modalities have a broad based potential for a variety of cancers and their indicated uses are being expanded. 

 In addition to the direct targeting to tumor cells via MAb therapy, the blockade of negative immunoregulatory mechanisms may contribute to the arsenal of immunotherapeutic treatments for cancer. The use of MAbs against immunosuppressant molecules, such as cytotoxic T lymphocyte antigen 4, has been explored and met with positive results in both human and murine models [81, 82]. Direct depletion of CD4+ CD25+ Tregs with low doses of an anti CD25 MAb has also been attempted and shown to be efficacious in murine models [83]. A reduction in Treg numbers may prove pivotal in disrupting tolerance to antigens within the tumor microenvironment, as this cell population is suspected to play a significant role in promoting immune tolerance to immunogenic determinants in many cancers [84]. Interestingly, the general population of Tregs can be broken down into distinct subpopulations, which may independently effect tolerance to tumor cells and express different markers for targeted depletion [84–86]. Paradoxically, other reports utilizing MAbs against the IL-2 receptor (CD25) have focused on the application of this approach as a mediator of generalized immune suppression [87]. In these scenarios, CD25 expressed on activated CD4+ and CD8+ T cells serves as the intended target for MAb-mediated depletion, as opposed to CD25 expressed on Tregs. Such discrepancies illustrate the complexities associated with immunotherapeutic strategies targeting common molecules that are expressed on disparate cell subsets.

## 6. Contraindications for Immune Mechanisms in Protecting Against Tumors

In addition to the role of the immune system in eliminating tumorigenic cells, it is well appreciated that the immune system can contribute, under certain circumstances, to the formation of tumors. Aside from the inherent difficulties in mounting robust immune responses to elements of self, both the innate and adaptive immune systems can directly confound mechanisms of tumor prevention and cure [88]. As mentioned previously, Tregs are thought to play an imperative role in mediating tolerance to some tumors. Although the mechanisms by which these cells mediate immune tolerance are still somewhat unclear, they may involve contact dependent inhibition of activated effector lymphocytes, as well as secretion of immunosuppressive cytokines such as IL-10 and transforming growth factor-*β* [89]. In experimental murine models of cancer and autoimmune disease, removal of Tregs has consistently been shown to enhance immune responses, resulting in inhibition of tumorigenic growth and exacerbation of autoimmune disease, respectively [90]. Furthermore, infiltrates of Tregs are commonly observed in sites of chronic viral disease, where they may function to inhibit immune responses to microbial pathogens in a capacity similar to that for tumors. 

 In addition to the various regulatory components of adaptive immunity that may disrupt immune responses to tumors, certain aspects of innate immunity appear to directly promote tumorigenesis under some circumstances. Chronic inflammation has long been regarded as a major contributing factor to the formation of tumors, and is often considered an important factor in the prognosis associated with certain cancers [91]. As indicated previously, chronic inflammation induced by microbial pathogens such as *H. pylori*, HBV, and HCV often correlates with tumor development in infected tissues. The constant tissue remodeling and angiogenesis associated with localized inflammation is apt to create a supportive environment for tumor formation and maintenance, while the actual process of oncogenesis may be accomplished by the release of DNA-damaging oxygen species and other toxic molecules from local leukocytes. Importantly, chronic inflammation may be supported by helper T cells and other components of adaptive immunity, which can activate resident leukocytes, such as macrophages, as well as secrete proinflammatory cytokines. Moreover, the ultimate role of inflammation in preventing versus protecting from tumor development deserves further assessment, although it is curious that long-term usage of certain antiinflammatory drugs, such as cyclooxygenase-2 inhibitors, can significantly reduce the risk of cancer [92].

## 7. Conclusions

The application of immunotherapeutic techniques to treat cancer is a vital and compelling pursuit of modern medicine. The increasing incidence of cancer in the western world demands continued evaluation of such techniques, and the development of new therapeutic strategies to combat malignant disease. A multitude of immunotherapeutic techniques, comprising efforts to exploit both active and passive immunity, are currently under investigation. Within the last decade, a small but growing body of therapeutic protocols representing vaccination, cytokine therapy, and MAb therapy have achieved FDA approval for the treatment of malignant disease. Interestingly, the most impressive clinical data has probably come from adoptive cellular therapy; however, at present, this approach has failed to proceed beyond clinical trials. One technique that may hold considerable promise for the future of cancer immunotherapy is vaccination. Indeed, the profound achievements of vaccination in controlling infectious disease have prompted a number of laboratories, including our own, to devote particular attention to the application of this approach to treat cancer. Moreover, a combination of current therapeutic strategies will likely be a key component in maximizing immune responses to various cancers, and in providing cancer patients with a comprehensive selection of treatment options. Perhaps in the foreseeable future, prudently crafted immunotherapeutics will overtake chemotherapy, radiation, and surgery as the dominant and less toxic strategy for treating malignant disease, and will provide cancer patients with effective treatment options for most, if not all, known human malignancies.

## Figures and Tables

**Table 1:** Examples of common tumor antigens.

Category	Antigen	Associated cancer types
Tumor-specific - viral	HPV: L1, E6, E7	Cervical carcinoma
	HBV: HBsAg	Hepatocellular carcinoma
	SV40: Tag	Malignant pleural mesothelioma

Tumor-specific - self	CDK-4	Melanoma
	*β*-catenin	Melanoma
	Caspase-8	Head/neck

CT antigen	MAGE-A1	Melanoma, myeloma, bladder, breast, prostate, lung, head/neck, esophageal, sarcoma
	NY-ESO-1	Melanoma, myeloma, bladder, breast, prostate, lung, head/neck, esophageal, sarcoma

Overexpression	MUC1	Breast, ovarian
	MUC13/CA-125	Ovarian
	HER-2/neu	Breast, melanoma, ovarian, gastric, pancreatic
	Mesothelin	Malignant pleural mesothelioma, ovarian, pancreatic
	PSMA	Prostate
	TPD52	Prostate, breast, ovarian

Differentiation	CEA	Colon
	Gp100	Melanoma
	MART-1/Melan-A	Melanoma
	Tyrosinase	Melanoma
	PSA	Prostate
	PAP	Prostate

Abbreviations: HPV, human papilloma virus; HBV, hepatitis B virus; SV40, simian virus 40; L, late gene; E, early gene; HBsAg, hepatitis B surface antigen; Tag, large tumor antigen; CDK, cyclin-dependent kinase; CT, cancer/testis; MAGE, melanoma-associated antigen; NY-ESO, New York esophageal squamous cell carcinoma; MUC, mucin; CA, cancer antigen; HER/neu, human epidermal receptor/neurological; PSMA, prostate-specific membrane antigen; TP, tumor protein; CEA, carcinoembryonic antigen; Gp, glycoprotein; MART/Melan-A, melanoma antigen recognized by T cells/melanoma antigen-A; PSA, prostate specific antigen; PAP, prostatic acid phosphatase.

**Table 2:** FDA approved monoclonal antibodies for use in cancer therapy.

Antibody	Target	Developer	Approved cancer treatments
Rituximab	CD20	IDEC Pharmaceuticals	Non-Hodgkin lymphoma
Trastuzumab	ErbB2	Genentech/UCLA	Breast
Gemtuzumab ozogamicin	CD33	Wyeth	Acute myeloid leukemia
Alemtuzumab	CD52	Genzyme Corporation	Chronic lymphocytic leukemia
Ibritumomab tiuxetan	CD20	IDEC Pharmaceuticals	Non-Hodgkin lymphoma
^131^I-tositumomab	CD20	Corixa	Non-Hodgkin lymphoma
Cetuximab	EGFR	ImClone Systems	Colorectal, head/neck
Bevacizumab	VEGF	Genentech	Colorectal
Panitumumab	EGFR	Amgen	Colorectal

Abbreviations: EGFR, epidermal growth factor receptor; VEGF; vascular endothelial growth factor.
